# Simple febrile seizure et causa bronchopneumonia with complex congenital heart disease

**DOI:** 10.21542/gcsp.2024.32

**Published:** 2024-08-01

**Authors:** Arga Setyo Adji, Syalomitha Claudia Stefanie Angel, Ronald Pratama Adiwinoto, Bambang Edi Suwito, Angela Puspitasari

**Affiliations:** 1 Faculty of Medicine, Hang Tuah University, Surabaya, Indonesia; 2 Department of Public Health, Hang Tuah University, Surabaya, Indonesia; 3 Department of Anatomy and Histology, Nahdalatul Ulama Surabaya University, Surabaya, Indoneisa; 4 Department of Emergency Medicine, Hang Tuah University, Surabaya, Indonesia

## Abstract

Complex congenital heart disease (CHD) in children does not often cause febrile seizures. A child with simple febrile seizures, bronchopneumonia, and complex congenital heart disease is discussed. The report analyzes the causes and proposes preventive measures for febrile seizures, bronchopneumonia, or both. A 2-year-old girl with complex congenital heart disease presented with simple febrile seizures possibly caused by bronchopneumonia. The child was severely malnourished (<-3SD), with a body weight of 7.5 kg and a height of 78 cm. Diagnostics revealed leukocytosis and neutropenia, with X-ray results showing abnormalities in the lungs and heart (ASD, VSD, and PDA). Treatment included diazepam 5 mg rectally for the first seizure and 1 mg IV for the second seizure, as well as paracetamol infusion 5 ml/4 h. Understanding simple febrile seizures triggered by bronchopneumonia in patients with complex congenital heart disease requires an integrated approach for patient management, including comprehensive care. The involvement of medical personnel is an important challenge in preventing recurrence and ensuring optimal patient outcome. Simple febrile seizures are likely caused by bronchopneumonia accompanied by complex congenital heart disease. Her recovery after prompt treatment with diazepam and symptomatic drugs emphasizes the importance of recognizing seizure triggers and managing febrile seizures in children with cardiac anomalies.

## Introduction

Febrile seizures associated with congenital cardiac anomalies represent convulsive occurrences observed during episodes of elevated body temperature in pediatric patients with preexisting structural abnormalities in the heart present since birth. When coupled with intricate congenital cardiac anomalies necessitating surgical intervention during the neonatal phase, their incidence becomes exceedingly uncommon^1,2^. Bronchopneumonia in children can lead to febrile seizures due to fever associated with lung infection. The child presented with seizures accompanied by fever, coughing, and difficulty expelling yellowish phlegm, which are symptoms consistent with bronchopneumonia. Fever triggers seizures in susceptible children, particularly within the age range in which febrile seizures are most common^3,4^.

In certain nations, febrile convulsions are the predominant etiology of seizures during infancy, manifesting at rates ranging from 2–5% in European and American juveniles. Nevertheless, the frequency appears to escalate in select Asian regions, such as Japan’s incidence rate of 7–10% and Guam’s incidence rate of 14%.

Indonesia, for instance, reports an approximate prevalence of 2.5 cases per 1000 live births afflicted by congenital heart defects (CHD)^5^. This suggests that certain Asian populations may have a higher incidence of febrile seizures than their counterparts in the US. Regarding gender differences, the incidence of febrile seizures appears to be unaffected by gender, indicating that both boys and girls develop febrile seizures at similar rates^6^. Although cohort studies reported no deaths among children with febrile seizures, the child death series indicated a 24.1% mortality rate associated with febrile seizures lasting 30 min or less^7^.

The categorization of febrile seizures, as per the National Collaborative Perinatal Project, involves two primary classifications: uncomplicated and intricate. Uncomplicated febrile seizures manifest in a generalized manner, endure for a duration of less than 15 min, manifest singularly within a 24-h timeframe, and culminate in the child’s restoration to a normative neurological state subsequent to the seizure episode. Conversely, intricate febrile seizures may exhibit focal characteristics, persist for durations exceeding 15 min, recur amidst the same febrile episode or within a 24-h interval, and potentially induce postictal neurological irregularities^8^.

In this case, we present a toddler diagnosed with a simple febrile seizure accompanied by bronchopneumonia, ventricular septal defect (VSD), atrial septal defect (ASD), or patent ductus arteriosus (PDA). This study was conducted at a tertiary hospital located in East Java, Surabaya. This report aims to not only analyze the causes of febrile seizures but also formulate improvement procedures to prevent their recurrence. Emphasizing the significance of reporting such cases, we recognize the importance of shedding light on the complexities of this medical scenario within the specific setting of the tertiary hospital in Surabaya.

### Case presentation

A 2-year-old girl presented to the Emergency Department with a fever of 39.5 °C, cough, and runny nose for two days. She was born *via* vaginal delivery, weighing 2250 g, with a current weight of 7.5 kg. She had a history of congenital heart disease, including atrial septal defect (ASD), ventricular septal defect (VSD), and patent ductus arteriosus (PDA), which was diagnosed at 1 year and 7 months of age, accompanied by a continuous pan-systolic murmur. TORCH screening for the child and her mother was completed, and the child’s immunization history was up-to-date. Regarding growth and development, at 1 year and 6 months of age, the child exhibited ”bluish skin/tet-spell” at the corners of her lips, fingertips, and toes. This episode prompted her admission to the hospital. At 2 years of age, she had just started crawling and could not form simple sentences.

During a three-day hospitalization period, the patient experienced a single episode of seizure activity on the subsequent day. The seizures manifested with a duration of three minutes, characterized by generalized convulsive movements accompanied by upward deviation of the eyes and loss of consciousness. Before the seizure, the child exhibited normal behavior, but post-ictal crying ensued.

Before the seizure, the child displayed symptoms of fever, cough, and runny nose while crying ensued post-ictally, with fever accompanying the seizure. The temperature recorded prior to the seizure was 39.5 °C. On admission, the patient had a fever of 38 °C, which temporarily subsided with antipyretic medication but reoccurred after approximately three hours. The fever was associated with a non-productive cough, yellowish sputum, and absence of symptoms such as rhinorrhea, sore throat, spasms, vomiting, or rash. The patient had no history of seizures, and this event was a singular occurrence, prompting a visit to the emergency department.

The patient had taken antipyretic medication alone and had not sought medical attention prior to hospitalization. Notably, the patient had a medical history of the use of oral spironolactone (8 mg) and lisinopril (0.8 mg) for congenital heart disease.

On physical examination, the patient presented with general malaise but demonstrated normal activity levels. Vital signs indicated a pulse rate of 112 beats per minute, respiratory rate of 27 breaths per minute, temperature of 39.5 °C, and oxygen saturation of 90%. The patient’s current weight was 7.5 kg, with a body length of 78 cm. Anthropometric measurements revealed severe undernutrition, marked by weight-for-height, weight-for-age, and height-for-age z-scores of −2.5, −2.5, and -3, respectively, and normocephalic head circumference. Respiratory examination revealed symmetrical chest expansion with normal chest morphology, and no deformities or retractions. Auscultation revealed bilateral vesicular breath sounds with coarse crackles in the lower lung fields, and normal heart sounds (S1 and S2) with a pansystolic murmur. Cardiovascular examination revealed palpable cardiac impulses at the fourth intercostal space bilaterally along the midclavicular lines, with percussion notes corresponding to the cardiac borders. Examination of the head, eyes, ears, nose, mouth, neck, and abdomen yielded unremarkable findings, as did examination of the extremities. Neurological examination findings were within normal limits.

On hematological examination (27/12/2023), a complete blood examination revealed leukocytosis (16.85 × 103/µL), neutropenia (42.10%), low Hb (10.80 g/dL) (Table 1), electrolyte and blood gas examination. within normal limits. Radiological examination revealed abnormalities in the lungs and the heart. Chest X-ray results (27/12/2023) showed an increase in BVP with visible cloudiness in both lungs, large and normal cast shape, sharp right and left phrenic costal sinuses, good right and left diaphragms, and good bones (Figure 2). In the history of cardiac ultrasound (05/05/2023), several abnormalities were found, including atrial septal defect (ASDII) with a diameter of 10 mm, a small perimembranous ventricular septal defect (VSD) with a diameter of four mm, and a moderately patent ductus arteriosus (PDA) with dimensions of two mm (Figure 1A–C).

**Table 1:** Hematology analysis results (month, year) (27-12-2023).

Para		Results	Unit	Reff. Ranges
1	WBC	16.85	10^3^/µL	4.00–12.00
2	Neu#	4.56	10^3^/µL	2.00–8.00
3	Lym#	5.36	10^3^/µL	0.80–7.00
4	Mon#	0.70	10^3^/µL	0.12–1.20
5	Eos#	0.21	10^3^/µL	0.02–0.80
6	Bas#	0.02	10^3^/µL	0.00–0.10
7	Neu%	42.10	%	50.0–70.0
8	Lym%	49.30	%	20.0–60.0
9	Mon%	6.50	%	3.0–12.0
10	Eos%	1.90	%	0.5–5.0
11	Bas%	0.2	%	0.0–1.0
12	IMG#	0.020	10^3^/µL	0.01–0.04
13	IMG%	0.200	%	0.16–0.62
14	RBC	3.27	10^6^/µL	3.50–5.20
15	HGB	10.80	g/dl	12.0–15.0
16	HCT	35.70	%	35.0–49.0
17	MCV	74.9	fL	73.0–101.0
18	MCH	24.7	Pg	23.0–31.0
19	MCHC	33.0	g/dl	26.0–34.0
20	RDW-CV	15.4	%	11.0–16.0
21	RDW-SD	42.9	fL	35.0–56.0
22	PLT	285	10^3^/µL	150–450
23	MPV	7.7	fL	6.5–12.0
24	PDW	15.0		15.0–17.0
25	PCT	0.212	%	0.108–0.282
26	P-LCC	28.0	10^3^/µL	30–90
27	P-LCR	9.7	%	11.0–45.0
*28	HFC#	****	10^3^/µL	
*29	HFC%	****	%	

**Figure 1A.**
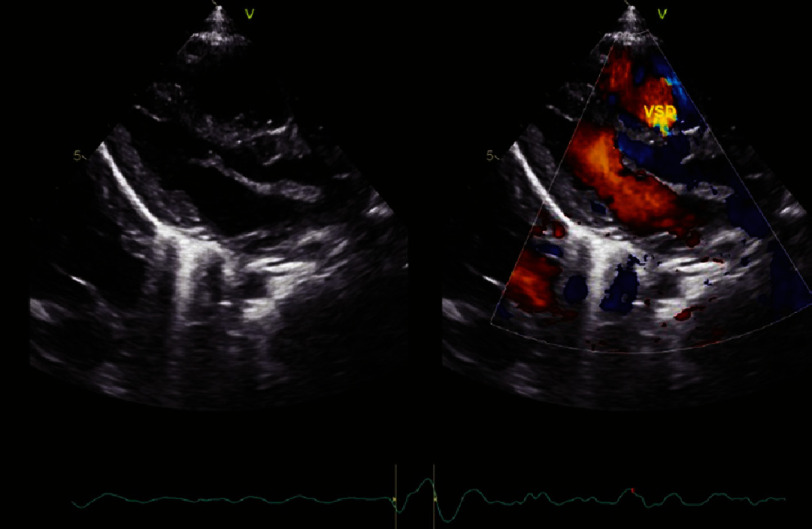
Small perimembranous ventricular septal defect (VSD).

**Figure 1B.**
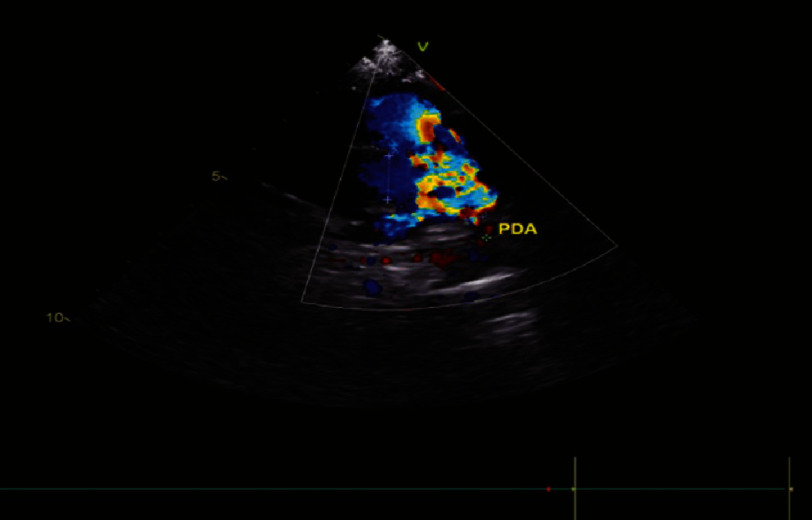
Moderate patent ductus arteriosus (PDA).

**Figure 1C.**
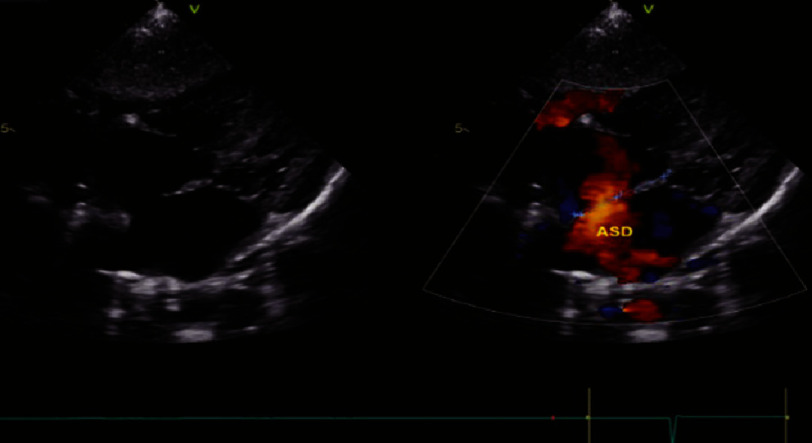
Moderate secundum atrial septal defect (ASD).

**Figure 2.**
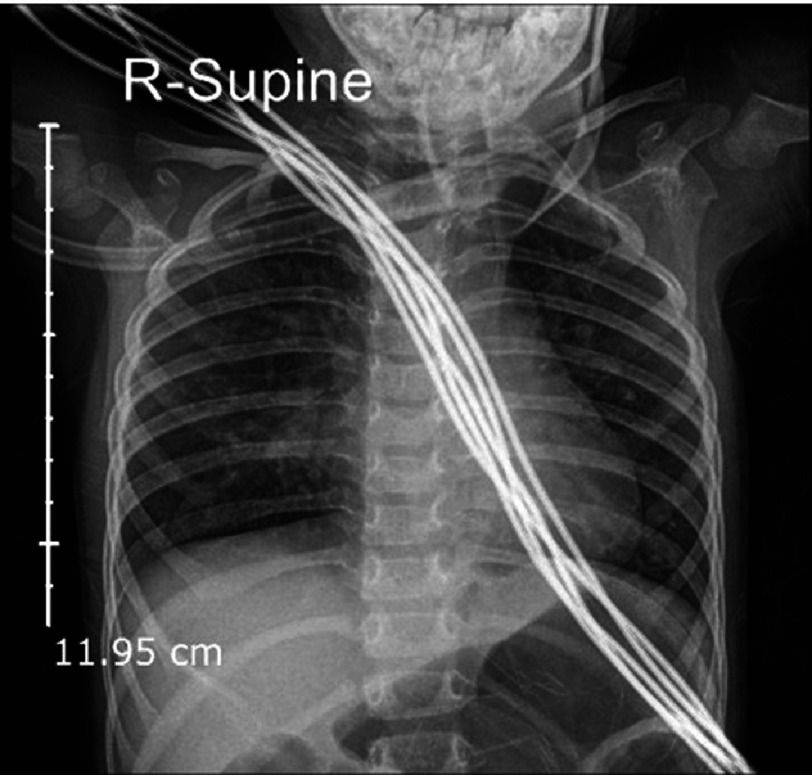
Chest AP radiology examination show BVP increases with the appearance of opacification in both lungs.

The patient got an infusion of D5 _1/4_ NS 750 cc/24 h, paracetamol 5 ml/4 h, diazepam supp 5 mg at the time of the first seizure in the ED, N-acetylcysteine 50 mg/8 h, albuterol nebul 1/2 respule + NS 2cc, ampicilin Na/sulbactam injection 4 × 200 mg, metamizole 3 × 80 mg injection, dexamethasone 3 × 0.7 mg, during seizures on day 2 given diazepam 1 mg IV bolus. After three days of treatment, clinical improvement was observed, and the patient was sent home.

### Case discussion

Febrile seizures are convulsions that can occur in toddlers, typically between 6 months and 5 years of age, and are caused by a fever, usually above 101 degrees Fahrenheit (38.3 degrees Celsius)^9^. Febrile seizures in children can be classified into two main types, namely simple (typical) febrile seizures and complex (atypical) febrile seizures. A simple febrile seizure, which is the most common variant, occurs when a child has one seizure event in 24 h, affecting the whole body and lasting less than 15 min. There is a possibility of recurrent simple febrile seizures if the child has had them before^10–12^. However, complex (atypical) febrile seizures may include signs such as irregular breathing, tooth biting or jaw locking, and a reduced level of consciousness. Seizures of this type may last more than 15 min or occur more than once in 24 h^10,12^.

Bronchopneumonia in children is a form of pneumonia that affects the bronchi and the surrounding lung tissue. It is generally caused by bacterial infection and can cause symptoms such as cough, fever, tachypnea (increased frequency of breathing), and difficulty breathing. Although bronchopneumonia can occur in children of different age groups, it is more common in infants and toddlers^13,14^. Children with CHD, especially those with cyanotic conditions, are at a higher risk of severe respiratory infections owing to their compromised cardiovascular and respiratory systems. Studies have shown that children with CHD have an increased incidence and recurrence of acute respiratory infections^15^. These infections often lead to extended hospital stays, increased morbidity, and a higher likelihood of complications such as seizures owing to the combined effects of hypoxia and systemic illness^16,17^. The vulnerability of these children to respiratory infections necessitates vigilant monitoring and prompt treatment to mitigate the risks associated with underlying heart conditions.

Complex congenital heart disease (CHD) in children refers to a group of heart defects that are present from birth and occur when the heart fails to develop properly during fetal development^18^. The incidence of congenital cardiac anomalies (CCA) in Indonesia is high. The predominant manifestations of CCA include ventricular septal defect (VSD), patent ductus arteriosus (PDA), atrial septal defect (ASD), and tetralogy of Fallot (TOF). Congenital cardiac anomalies exist from birth and are capable of altering cardiac structure and functionality, thereby engendering diverse clinical manifestations and potential morbidities in pediatric patients^19,20^. The prevalence of critical congenital heart disease (CCHD) in Indonesia is 6 out of every 1000 live births, and positive CCHD screening occurs in 0.6% of newborns^21^. Another study, published in 2017, predicted that the prevalence of births with moderate and severe disabilities would be approximately 1.5 per 1000 live births for each group^22^.

Children with congenital heart disease (CHD) are at a higher risk of experiencing seizures, especially those with critical CHD, owing to the potential consequences of severe hypoxia and hypoperfusion associated with their underlying condition^23^. However, it is important to clarify that CHD itself does not directly cause seizures; instead, complications, such as hypoxia and hypoperfusion, increase the risk. Additionally, the long-term neurological effects of recurrent hypoxia or hypoperfusion in CHD patients can significantly contribute to seizure risk over time. Recurrent episodes of hypoxia and hypoperfusion can lead to chronic brain injury, which manifests as various neurodevelopmental disorders including epilepsy. Studies have shown that children with CHD who experience frequent hypoxic episodes are more likely to develop neurological complications, such as cognitive deficits, developmental delays, and seizures^24,25^. These long-term effects highlight the importance of continuous monitoring and early intervention to manage hypoxia and hypoperfusion in children with CHD and mitigate the associated neurological risks. The increased threat posed by bronchopneumonia, a type of pneumonia affecting the bronchi and adjacent lung tissues, is attributed to the potential for respiratory compromise in children with CHD^13^.

The risk of seizures in children with congenital heart disease(CHD) varies significantly between those with cyanotic and acyanotic conditions. Infants with cyanotic CHD, characterized by low blood oxygen levels, are at a higher risk of neurological complications, including seizures. This increased risk is primarily due to chronic hypoxia and associated brain injury. Based on the case presentation, the patient exhibited symptoms consistent with cyanotic CHD, such as “bluish skin/tet-spell” episodes, indicating persistently low oxygen levels, further supporting the diagnosis of cyanotic congenital heart disease. In contrast, children with acyanotic CHD, which generally maintains normal oxygen levels, experience a different set of risks predominantly related to hemodynamic instability rather than direct hypoxic damage^26,27^. Additionally, metabolic disturbances and inflammation have been observed to be more severe in cyanotic CHD, potentially contributing to higher seizure susceptibility^28,29^. These findings underscore the need for tailored monitoring and intervention strategies for different CHD subtypes to mitigate the risks of seizures and other neurological sequelae. Huisenga et al. (2020) found that children with CHD have an increased risk of neurological complications, including seizures, with a risk ratio (RR) of 2.34 (95% CI [1.90–2.88]), and Jerrell et al. (2015) found that children with CHD are more likely to experience seizures compared to their peers, with an odds ratio (OR) of 2.00 (95% CI [1.66–2.41]). The study also noted that children who underwent cardiac and non-cardiac surgical interventions had a significantly increased risk of developing neurological conditions, including seizures^25,30^.

Fever (elevated body temperature) is a multifaceted physiological reaction to infection or inflammation. This response is initiated by the release of endogenous pyrogenic agents, notably cytokines, which activate prostaglandin synthesis within the hypothalamus. Consequently, there is an upward adjustment in the body’s thermoregulatory setpoint. Fever predisposes children to febrile seizures^31^. Fever can cause seizures *via* several mechanisms. One of these is the lowering of the seizure threshold in the immature cells. In addition, dehydration caused by fever can result in electrolyte disturbances and disruption of cell membrane permeability. Increased basal metabolism due to fever can also lead to the build-up of lactic acid and CO2, which can potentially damage the neurons. In addition, fever increases blood flow to the brain (CBF), which can increase the demand for oxygen and glucose, leading to impaired ion flow in and out of cells^32,33^.

The treatment of febrile seizures in children aims to ensure safety during seizures and to identify and address the cause of fever. The use of antipyretic drugs such as acetaminophen and ibuprofen has been shown to be effective in reducing the risk of febrile seizures if administered in the early stages of the illness. Immediate action in a child experiencing febrile seizures involves emergency stabilization with the ABCDE (Airway, Breathing, Circulation, Disability, and Exposure/Elimination) approach^34^.

According to previous research, although diazepam was not initially recommended for managing febrile seizures, it has proven effective in reducing the risk of recurrent febrile seizures when administered intermittently. In a controlled trial, oral diazepam at a dose of 0.33 mg/kg per dose every 8 h during febrile illness was found to be effective in preventing recurrent febrile seizures. The administration of oral diazepam may reduce the number of febrile seizures occurring during each febrile episode, and some practitioners may prescribe rectal diazepam, especially for patients experiencing prolonged febrile seizures, to prevent future episodes of febrile status epilepticus. Benzodiazepines, including diazepam, have the potential to cause side effects, such as lethargy, drowsiness, ataxia, and respiratory depression, and may potentially obscure the development of infection in the central nervous system^34–36^. In the previous drug history, the patient received oral administration of 0.8 mg of spironolactone and 8 mg of lisinopril. From this statement, it is known that spironolactone and lisinopril are used to treat children’s heart failure, including congenital heart disease. Spironolactone reduces the cardiovascular effects of aldosterone, whereas lisinopril antagonizes RAAS, reducing intravascular volume and salt retention. Both drugs increase cardiac output and remodeling, especially in young patients with CHD^37^.

The patient was a 2-year-old girl, consistent with the theory that febrile seizures in children occur between the ages of six months and five years. Febrile seizures are reported to be more common in boys than girls^38^. In the history of seizures accompanied by fever, with a seizure duration of 3 min, the seizure occurred once a day. This condition aligns with the criteria for the diagnosis of simple febrile seizures because there is one seizure in less than 24 h. Data obtained from anamnesis indicate that the patient presented with complaints of cough, accompanied by fever, and a runny nose for the past 2 days. In this case, bronchopneumonia was suspected to be the underlying cause of febrile seizures in the child.

### What we have learned?

In this case SFS with complex CHD et causa bronchopneumonia, the patient is a 2-year-old girl with a history of complex congenital heart disease, including ventricular septal defect, atrial septal defect, and patent ductus arteriosus, who presented with a febrile seizure triggered by bronchopneumonia. This case highlights the increased risk of febrile seizures in children with congenital heart disease. It underscores the importance of managing fever and the underlying cause to prevent recurrent seizures, with treatments such as antipyretic medication, antibiotics, and supportive measures being effective. The patient’s clinical improvement after three days of treatment demonstrates the effectiveness of these interventions. This case contributes to our understanding of the relationship between congenital heart disease, febrile seizures, and the role of underlying infections like bronchopneumonia in triggering such events.
